# One third of physicians discuss exit strategies with patients with amyotrophic lateral sclerosis: Results from nationwide surveys among German and Polish neurologists

**DOI:** 10.1002/brb3.3243

**Published:** 2024-01-06

**Authors:** Krzysztof Barć, Julia Finsel, Olga Helczyk, Susanne Baader, Helena Aho‐Özhan, Albert C. Ludolph, Dorothée Lulé, Magdalena Kuźma‐Kozakiewicz

**Affiliations:** ^1^ Department of Neurology University Clinical Centre of Medical University of Warsaw Warsaw Poland; ^2^ Department of Neurology University of Ulm Ulm Germany; ^3^ Department of Neurology Medical University of Warsaw Warsaw Poland; ^4^ Neurodegenerative Diseases Research Group Medical University of Warsaw Warsaw Poland; ^5^ German Centre of Neurodegenerative Diseases (DZNE) Ulm Germany

**Keywords:** amyotrophic lateral sclerosis, end‐of‐life care, euthanasia, life‐terminating strategies, physician‐assisted suicide, physicians

## Abstract

**Objective:**

This paper examines neurologists’ approaches to exit strategies (ESs), such as euthanasia and physician‐assisted suicide, in patients with amyotrophic lateral sclerosis (PALS) in two European countries.

**Methods:**

In a nationwide anonymous survey, we collected responses from 237 Polish and 228 German neurologists, focusing on their practices and beliefs about ESs, as well as their viewpoints on life‐sustaining measures (LSMs) (percutaneous endoscopic gastrostomy, non‐invasive, and invasive ventilation). To analyze the data, we employed statistical methods, including Mann–Whitney *U*, Kruskal–Wallis, chi‐square tests, Spearman's rank correlation, and multiple regression analysis.

**Results:**

One third of the neurologists initiated the discussion about ESs with PALS. Half were ready to have this conversation upon patient's request. Age, gender, religiousness, and nationality were closely associated with this approach. One in 9 neurologists received a request to terminate an LSM, whereas 1 in 10 to implement an ES. German neurologists and palliative care trainees acquired both demands more commonly. Neurologists quoted a low quality of life, decreased mood, and being a burden to the family/closest ones as primary reasons for a wish to hasten death among PALS. Although the majority expressed a willingness to terminate an LSM at a request of the patient, most opposed the legalization of euthanasia. Younger and less religious individuals were more likely to favor accepting euthanasia.

**Conclusion:**

Neurologists vary significantly in their approaches to terminal care. Complex relationships exist among personal indices, shared beliefs, and current practices.

## Graphical abstract

One third of neurologists initiate discussions on exit strategies with ALS patients, whereas half of them is ready to have such conversations. One in 9 neurologists have been asked by ALS patients to withdraw life‐sustaining measures, and 1 in 10 to implement an exit strategy. There are complex relationships among a neurologist's personal background, knowledge, shared beliefs, and current practices regarding exit strategies.

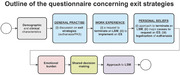

## 1 INTRODUCTION

Individuals with rapidly progressive fatal diseases such as amyotrophic lateral sclerosis (ALS) may have different priorities and treatment expectations (Chen et al., 2020; Chio et al., 2022; De Jongh et al., 2022; Masrori & Van Damme, 2020; Spittel et al., 2021; Tornese et al., 2022). Some prioritize life extension, whereas others may place greater emphasis on maintaining autonomy (Mohammed et al., 2020). In extreme cases, individuals may wish to hasten death due to the burden of progressive physical and psychosocial suffering (Borasio & Voltz, 1998).

Euthanasia and physician‐assisted suicide (PAS) are highly debated topics around the world, posing emotional and ethical challenges. Actions directly intended to end a patient's life have been legalized in few countries (Table 1) (Emanuel et al., 2016; Oliver & Turner, 2010) and are subject to specific requirements. These safeguards were designed not only to prevent potential overuse of these methods but also to differentiate the true desire for euthanasia or PAS from inadequate palliative care, unrecognized depression, or a financial burden (Bascom & Tolle, 2002; Emanuel & Battin, 1998; Ganzini & Block, 2002).

**TABLE 1:** Countries where euthanasia and/or physician‐assisted suicide (PAS) are law‐accepted procedures (as of July 2022).

Euthanasia	PAS
▪ Australia (Victoria and Western Australia) ▪ Belgium ▪ Canada ▪ Colombia ▪ Luxembourg ▪ The Netherlands ▪ Spain	▪ Australia (Victoria and Western Australia) ▪ Austria ▪ Belgium ▪ Canada ▪ Finland ▪ Germany ▪ Italy ▪ Luxembourg ▪ The Netherlands ▪ New Zealand ▪ Spain ▪ Switzerland ▪ US (10 states: CA, CO, HI, ME, NJ, NM, OR, VT, WA, DC, MT)

Abbreviations: CA, California; CO, Colorado; DC, District of Columbia; HI, Hawaii; ME, Maine; MT, Montana; NJ, New Jersey; NM, New Mexico; OR, Oregon; US, United States; VT, Vermont; WA, Washington.

*Source*: Britannica; ProCon (https://euthanasia.procon.org/euthanasia‐physician‐assisted‐suicide‐pas‐around‐the‐world).

The existing guidelines for palliative care in ALS do not cover exit strategies (ESs), encompassing facets of assisted dying such as euthanasia and PAS, attributed to a scarcity of clinical trial data (Bede et al., 2011; Hui et al., 2020; Oliver et al., 2016). Before formulating such recommendations, it is crucial to delve into significant consideration, including patient well‐being, care objectives, and expectations held by both patients and their caregivers. The perspectives of physicians on these matters bear significant weight as they typically initiate discussions on various treatment‐related topics and shape consultation content based on their experience and the patient's condition. Therefore, understanding the factors driving current practice and physicians’ attitudes toward end‐of‐life care in ALS is essential in comprehending their potential influence on patients’ decisions. In addition to analyzing the physician's knowledge, professional and personal experiences, as well as personal beliefs, we hypothesize that other domains might play role. One such domain could be anticipated outcomes of available treatment options, including the use of life‐sustaining measures (LSMs) like percutaneous endoscopic gastrostomy (PEG) and non‐invasive or invasive ventilation (NIV and IV) (Barć & Kuźma‐Kozakiewicz, 2020). Previous studies have demonstrated a connection between physicians’ expectations of patient well‐being with ventilation and their treatment preferences (Barć et al., 2022; Uhlmann, 1991). Additionally, initiating discussions on ESs can be challenging for some physicians (Faull et al., 2014); hence, their level of self‐confidence may influence the likelihood of addressing this topic in clinical care. Increased self‐confidence has been shown to boost the chances of engaging in discussions about medical matters (Barć & Kuźma‐Kozakiewicz, 2020; Goto et al., 2021; Martin et al., 2016; Ruffell et al., 2013). Moreover, the emotional burden often experienced by physicians caring for patients with short survival times may result in avoidance of end‐of‐life care conversations (Faull et al., 2014; Schuurmans et al., 2021).

In addition to the aforementioned aspects, variances in sociocultural and national legal settings may further shape the perceptions and practices regarding ESs among neurologists. This study focused on two neighboring European countries—Germany and Poland, where euthanasia is illegal. However, a notable divergence exists between the two countries; assisted suicide conducted by the patients’ kin (but not physicians) has been decriminalized in Germany since 2015, unlike in Poland (Horn, 2020). In 2020, the German Federal Constitutional Court rejected the 2015 law that prohibited company‐organized (business‐like) assisted suicide, considering it unconstitutional. Thus, although the Court judgment has opened an indirect path to acceptance of PAS in Germany, the corresponding administrative law has not been issued yet. A second difference is that Germany grants the right to withdraw IV or tube‐feeding in response to a patient's request. In contrast, such an action in Poland, if the treatment is indispensable for the patient's survival, is considered assisted suicide and is inconsistent with the Medical Ethics Code (Chańska, 2013). Despite the emphasis on upholding patient dignity and privacy at every stage of treatment as stipulated by the Polish Medical Ethics Code and the Patient Rights Protection Act and Patient Ombudsman (*Ustawa o ochronie praw pacjenta i Rzeczniku Praw Pacjenta*), a significant consideration must be given to the potential extension of survival when PEG or IV is implemented, and the consequent loss of life following their discontinuation. Hence, the decision to terminate these interventions is not allowed to be employed by the physician in people affected by ALS in Poland.

Therefore, the aim of our study was to (i) determine neurologists’ (a) general approach to discussing ESs with ALS patients (PALS), (b) clinical experiences with issues concerning ESs, (c) personal attitudes toward ESs, as well as (ii) to establish determinants of attitudes toward ESs, and (iii) examine country‐specific differences between Germany and Poland.

## 2 METHODS

### 2.1 Data collection

**FIGURE 1:**
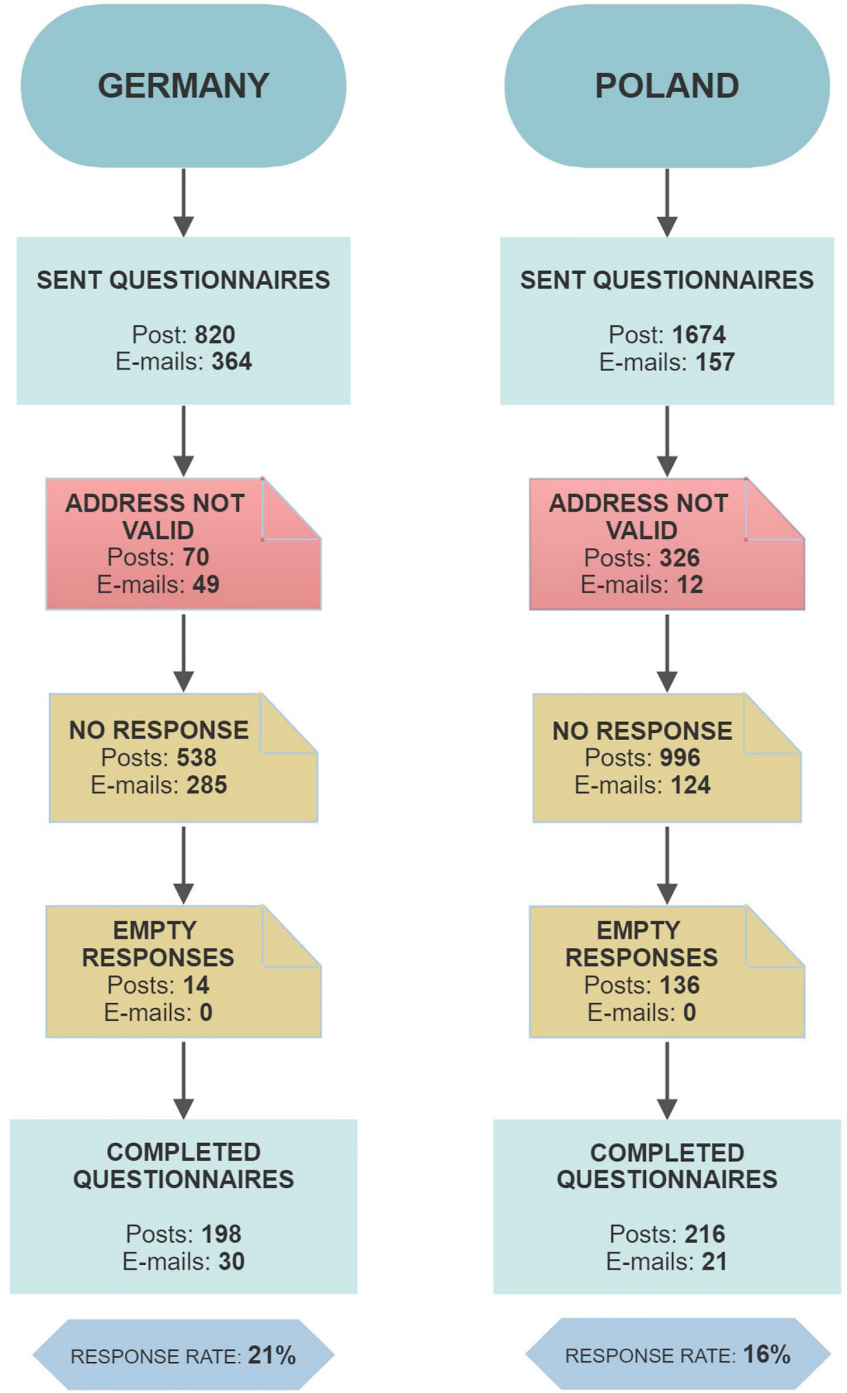
Data collection for the study.

Between June 2016 and April 2019, we sent out 3015 questionnaires by traditional or electronic mail to evenly selected neurologists from all provinces in Germany and Poland (Figure 1). We gathered information on physicians’ names, places of work, and e‐mail addresses (if available) from several online platforms, including www.jameda.de, www.arzt‐auskunft.de, www.znanylekarz.pl, and www.mp.pl/lekarz. We searched each platform using relevant keywords (e.g., “Nervenarzt,” “Neurologe,” “neurolog,” and “lekarz neurolog”) and applied inclusion criterion of specialization in neurology to select the data. We received 237 self‐completed questionnaires from the Polish participants and 228 from the German respondents (response rates: 16% and 21%, respectively). The reasons for returned envelopes (n=457) were most commonly invalid addresses (e.g., not working at the given address anymore) or temporary absence (e.g., maternity leave or holiday break). The primary reason for sending back a blank questionnaire (*n* = 150) was a lack of or little experience in ALS.

### 2.2 Questionnaire

**FIGURE 2:**
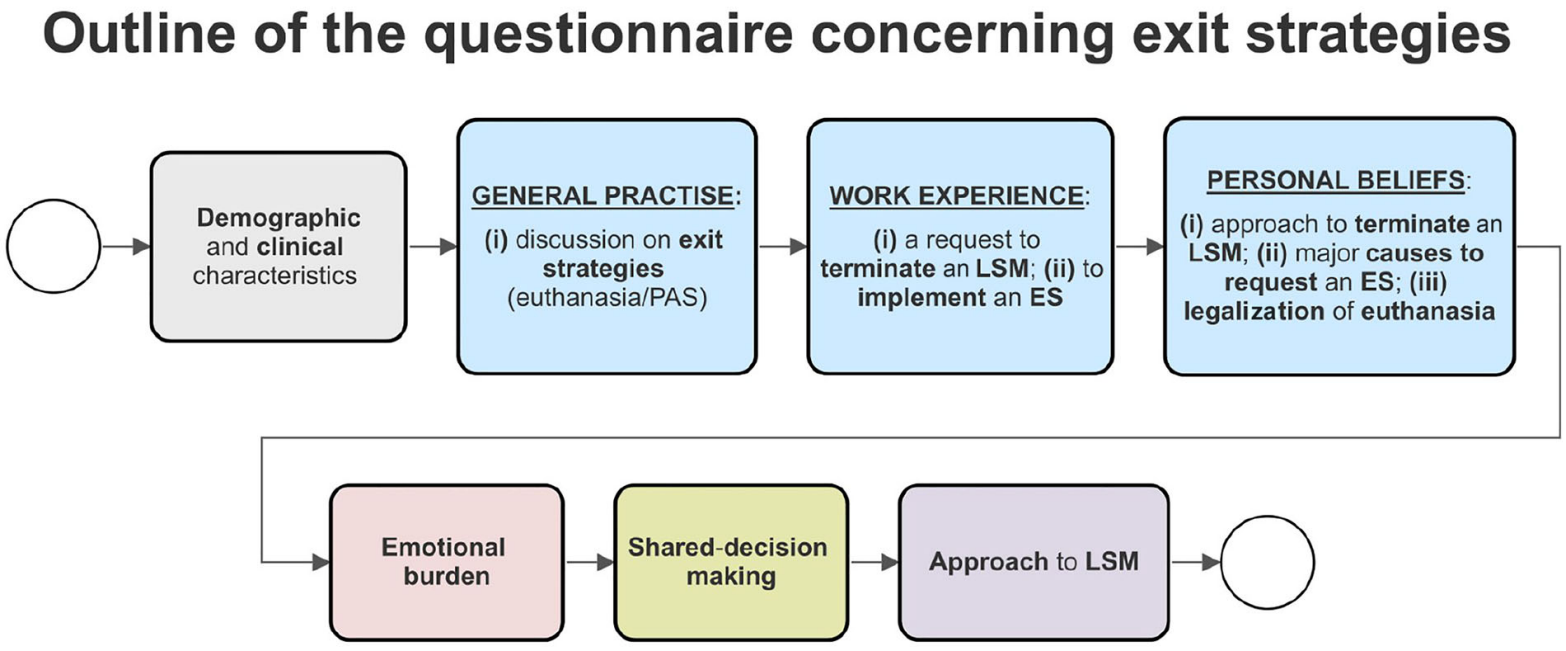
Outline of the questionnaire. ES, exit strategy; LSM, life‐sustaining measures.

We asked physicians to fill out an anonymous questionnaire about their *general practice*, *experience*, and *attitudes toward ES*, as detailed later. For further analysis, we also requested responders to declare their opinions on the *LSM*, *emotional burden* and *shared decision‐making*. In addition, we collected data on demographics (*age*, *gender*, *relationship status*, *having children*, and *religiousness*) and professional experience (*number of ALS patients seen per month, number of years of experience in ALS*, *completed palliative care training (PCT)*, and *participation in ALS research)*. Figure 2 shows an outline of the questionnaire.

A comprehensive explanation of the questionnaire can be found in Supporting Information A.

### 2.3 Statistical analysis

Chi‐square and Student's *t* tests were used as appropriate for demographic and professional experiences comparisons between the two countries. To explore the determinants of demographic and professional experience, binary, multinomial, and ordinal regression models were applied. In these analyses, we used an experience rate (ExpR) as a controlling variable due to a strong correlation between *the number of patients seen per month* and *years of experience in ALS* for the multiple regression models. This ExpR was calculated as the *number of PALS seen per month on average* × *years of experience in ALS*.

Nonparametric tests (chi‐square test, Mann–Whitney U or Kruskal–Wallis test, and Spearman's correlation coefficients) were used to investigate the nominal, ordinal, and continuous variables, respectively. Additionally, we applied Bonferroni's post hoc analysis for the question about the *general practice* to investigate between‐group differences. Analyses were performed in the software SPSS v28.0.1.0 (IBM SPSS Statistics).

## 3 RESULTS

### 3.1 Cohort characteristics

Of the 465 surveyed neurologists, the majority (59.8%) were middle‐aged (40–59 years old) with a male‐to‐female ratio of 1.12%, 72.6% had over 10 years of experience in caring for PALS, and 78.9% would see one‐to‐three ALS patient(s) per month on average. In the German group (228/465, 49%), there were more males and individuals trained in PCT, a lower median value of self‐reported religiousness, and a higher prevalence of ALS research participants compared to the Polish group (237/465, 51%) (Table 2).

**TABLE 2:** Neurologists’ characteristics.

	Entire group	German group	Polish group	*p‐Value*
**Gender**, %				
Male	52.86	62.74	42.79	
Female	47.14	37.26	57.21	*<*.*001* ^a^
**Age**, %				
<30	3.38	3.67	3.08	
30–39	14.83	13.76	15.86	
40–49	26.97	30.28	23.79	
50–59	32.81	35.32	30.40	
60–69	17.53	14.22	20.70	
>70	4.49	2.75	6.17	*.123*
**In a relationship**, %				
Yes	85.2	87.1	83.3	
No	14.8	12.9	16.7	*.264*
**Children**, %				
Yes	83.07	80.93	85.09	
No	16.93	19.07	14.91	*.244*
**Religiousness**, %				
Non‐believing	19.41	23.15	15.86	
Quite believing	34.09	46.3	22.47	
Believing	38.15	26.39	49.34	
Deeply believing	8.35	4.17	12.33	*<.001* ^a^
**How many patients seen per month**, %				
<1	15.95	17.11	14.83	
1–3	78.88	75.00	82.63	
4–10	34.48	5.70	1.27	
>10	1.72	2.19	1.27	*.547*
**How many years of experience in ALS**, %				
<1	6.04	4.82	7.20	
1–5	8.62	8.77	8.47	
5–10	12.72	12.72	12.71	
>10	72.63	73.68	71.61	*.542*
**Palliative care training**, %				
Yes	20.26	12.28	27.97	
No	79.74	87.72	72.03	*<.001* ^a^
**Taking part in ALS research**, %				
Yes	15.7	19.7	11.9	
No	84.3	80.3	88.1	*.020* ^a^

Abbreviation: ALS, Amyotrophic Lateral Sclerosis.

^a^Significant at an alpha‐level of p < .05.

Supporting Information B, Tables II and III summarize all responses and their determinants (demographic and clinical background).

### 3.2 General practice

#### 3.2.1 “Do you discuss exit strategies (euthanasia or PAS) with ALS patients?”

Nearly one third of neurologists (30.2%) discussed ES with PALS on their own initiative (5.3% initiated the discussion at the time of diagnosis, whereas 24.9% at the advanced disease stage), 47% engaged in the conversation only upon the patient's request, whereas 22.8% never attempted the discussion.

*Demographic and clinical predictors*: Neurologists *initiating* the discussion on ES were *younger*, predominantly *male* and showed *lower values of self‐reported religiousness* compared to groups addressing these issues *at the patient's request* or those who *never* did so (Table 3).

**TABLE 3:** Neurologists’ clinical and demographic determinants of the clinical approach toward the discussion of exit strategies with amyotrophic lateral sclerosis (ALS) patients.

	Chi‐square	df	*p*‐Value		
			All groups	Initiate (vs. when asked)	Initiate (vs. never)
Demographic					
**Age**	16.209	2	.003^a^	.023^a^	.001^a^
**Gender**	16.322	2	<.001^a^	.018^a^	.142
**Religiousness**	9.470	2	.025^a^	.032^a^	.015^a^
**In a relationship**	20.600	2	.225	.281	.538
**Having children**	2.568	2	.365	.439	.473
**Nationality**	40.483	2	<.001^a^	.202	<.001^a^
*Clinical*					
**Experience rate**	0.802	2	.779	.977	.520
**PCT**	2.138	2	.447	.926	.293
**ALS research**	4.054	2	.197	.537	.215

Abbreviation: PCT, palliative care training.

^a^Significant at an alpha‐level of p < .05.

*A link between LSM and ES*: Favorable estimation of the well‐being of PALS using LSM was related to a higher prevalence of initiating the discussion on ES (Table 4).

**TABLE 4:** Relationship between neurologists’ beliefs on life‐sustaining measures (LSMs) and clinical practice in discussing the exit strategies with patients with amyotrophic lateral sclerosis (PALS).

	“Do you discuss exit strategies (*euthanasia* or *PAS*) with PALS?”						
					*Post hoc test*		
	Initiate (1)	On a request (2)	Never (3)	*p‐Value*	1–2	1–3	2–3
“How do you estimate the QoL/depressiveness in ALS patients using a particular LSM?”							
**QoL on PEG**, median [Q_1_–Q_3_]	1 (−1, 2)	0 (−2, 2)	0 (−2, 2)	*.002* ^a^	*.81*	*.004* ^a^	*.493*
**Depressiveness on PEG**, median [Q_1_–Q_3_]]	5 (3, 7)	5 (3, 7)	6 (4.25, 9)	*.004* ^a^	*1.0*	*.006* ^a^	*.014* ^a^
**QoL on IV**, median [Q_1_–Q_3_]	−2 (−4, 0)	−2 (−4, −1)	−3 (−4, −1)	.*110*	*–*	*–*	*–*
**Depressiveness on IV**, median [Q_1_–Q_3_]	7 (4, 8)	7 (5, 8)	8 (5, 10)	.*009* ^a^	*1.0*	*.012* ^a^	*.025* ^a^
**QoL on NIV**, median [Q_1_–Q_3_]	1 (−1, 2)	0 (−2, 2)	−1 (−2, 2)	*.006* ^a^	*.168*	*.001* ^a^	*.118*
**Depressiveness on NIV**, median [Q_1_–Q_3_]	5 (3, 7)	5 (3, 7)	6 (4.75, 9)	*.004* ^a^	*1.0*	*.004* ^a^	*.021*
“Do you consider each LSM beneficial to ALS patients?”							
**PEG**, %				*.600*	–	–	–
Yes	60.8	53.2	71.4				
Rather yes	35.2	36.3	24.5				
Rather no	2.4	8.5	1				
No	1.6	2.0	3.1				
**IV**, %				*.020* ^a^	*1.0*	*.020* ^a^	*.088*
Yes	21	14.7	36.5				
Rather yes	36.3	34.5	36.5				
Rather no	34.7	41.6	16.7				
No	8.1	9.1	10.4				
**NIV**, %				*.314*	–	–	–
Yes	58.2	47.5	58.2				
Rather yes	36.9	43.5	31.6				
Rather no	4.1	6.5	7.1				
No	0.8	2.5	3.1				
“Would you decide for an LSM yourself in case of indications?”							
**Opt for PEG**, %				*.099*	–	–	–
Yes	36.1	37.7	48.5				
Rather yes	45.9	41.7	33				
Rather no	10.7	10.6	8.2				
No	7.4	10.1	10.3				
**Opt for IV**, %				*<.001* ^a^	*.386*	*<.001* ^a^	*.037*
Yes	7.4	9.6	14.6				
Rather yes	22.3	22.3	32.3				
Rather no	31.4	34.5	27.1				
No	38.8	33.5	26.0				
**Opt for NIV**, %				*.006* ^a^	*.285*	*.005* ^a^	*.448*
Yes	42.1	38.2	46.4				
Rather yes	43	45.2	36.1				
Rather no	6.6	9.0	9.3				
No	8.3	7.5	8.2				

Abbreviations: PAS, physician‐assisted suicide; QoL, quality of life; ALS, Amyotrophic Lateral Sclerosis; PEG, percutaneous endoscopic gastrostomy; IV, invasive ventilation; NIV, non‐invasive ventilation.

^a^Significant at an alpha‐level of p < .05.

*Shared‐decision approach/emotional burden*: Neurologists who *initiated* discussions, as well as those who engaged in conversations *only upon the patient's request*, exhibited a more positive approach toward advising patients on therapeutic matters (post hoc *p* < .001 and *p* < .001, respectively) and reported lower levels of emotional distress (post hoc *p* = .042 and *p* = .023, respectively), in comparison to their counterparts who *never* attempted ES discussions.

Cross‐*national differences*: Although no significant difference was observed in the *initiation* of ES discussions between German and Polish neurologists, German physicians more frequently started the discussion *only upon the patients’ request* (56.6% vs. 37.0%, respectively; post hoc *p <* .001), whereas the Poles more often *did not have* such discussion (10.5% and 35.5%, respectively; post hoc *p <* .001).

### 3.3 Personal experience

#### 3.3.1 “Have you ever been asked to terminate a life‐sustaining measure?”

Eleven percent of the surveyed neurologists declared to have ever been asked to cease an LSM.

*Demographic and clinical predictors*: Completion of PCT (odds ratio [OR]: 4.72 [95% confidence interval [95% CI], 4.72–9.8], *p <* .001) was significantly associated with having received a request to stop an LSM (Table 5).

**TABLE 5:** The relationship between the training in palliative care and the clinical experience with exit strategies (ESs) among German and Polish neurologists caring for amyotrophic lateral sclerosis (ALS) patients.

	Germany			Poland		
*Neurologists’ experience*	**PCT(+)**	**PCT(−)**	*p‐Value*	**PCT(+)**	**PCT(−)**	*p‐Value*
*“Ever asked by a patient about terminating LSM?”* ^a^	Yes: **37.5%**	Yes: **10.2%**	*<.001^b^*	Yes: **18.5%**	Yes: **5.0%**	*<.001* ^b^
*“Ever asked by a patient about implementing an ES?”* ^a^	Yes: **16.3%**	Yes: **8.3%**	*.022^b^*	Yes: **7.8%**	Yes: **1.3%**	*.011* ^b^
*“Would you cease an LSM on a patient's request?”* ^c^	Yes: **33.3%** Rather yes: **51.9%**	Yes: **13.7%** Rather yes: **58.4%**	*.049^b^*	Yes: **10.8%** Rather yes: **20.0%**	Yes: **12.0%** Rather yes: **26.6%**	*.560*

Abbreviations: LSM, life‐sustaining measures; PCT(−), the group of neurologists with no training in palliative care; PCT(+), the group of neurologists who completed training in palliative care.

^a^ Possible answers: *yes*; *no*.

^b^ Significant at an alpha‐level of p < .05.

^c^ Possible answers: *yes*; *rather yes*; *rather no*; *no*.

*Cross‐national differences*: German neurologists more frequently obtained the question to cease an LSM (OR: 2.47 [95% CI, 1.13–5.38], *p =* .023—“Yes”/“No”: 13.4%/86.6% vs. 8.8%/91.2%). Among the Polish physicians, only those who perceived quality of life (QoL) to be lower in people using PEG were more likely to be asked to withdraw an LSM (*p =* .027).

#### 3.3.2 “Have you ever been asked to implement an exit strategy?”

About 1 in 10 neurologists (9.9%) reported having been asked to implement an ES.

*Demographic and clinical predictors*: Completion of PCT (OR: 3.75 [95% CI, 1.69–8.33], *p =* .001) showed a significant association with being asked to deploy an ES.

*A link between LSM and ES*: Lower values of estimated depressiveness in PALS using NIV (*p* = .025), IV (*p* = .04), and PEG (*p* = .008) characterized neurologists who had been asked to implement an ES. In addition, a similar relation was found in individuals who were more likely to indicate IV as a non‐beneficial measure for PALS (*p* = .005).

*Shared‐decision approach/emotional burden*: The neurologists who have been asked to execute an ES reported lower values of emotional distress (*p =* .003) and a more favorable approach toward advising PALS on medical choices (*p <* .001).

*Cross‐national differences*: German neurologists received the above request more often compared to the Polish (OR: 7.19 [95% CI, 2.82–18.52], *p <* .001—“Yes”/ “No”: 17%/83% vs. 3.1%/96.9%). Exclusively in the Polish group, participation in ALS research had a significant association with reaching this question.

### 3.4 A personal approach

#### 3.4.1 “Would you terminate a life‐sustaining measure if asked by a patient?”

Over half of the neurologists (54.6% [13.8%, “Yes”; 40.8%, “Rather Yes”]) expressed a willingness to withdraw an LSM on the patient's request.

*Demographic and clinical predictors*: Neurologists of *younger age* (CI 95%, .441–.086, *p =* .004) and *lower values of self‐reported religiousness* (CI 95%, .804–.356, *p <* .001) showed a more favorable approach toward ceasing an LSM.

*A link between LSM and ES*: Favorable approach toward terminating an LSM correlated inversely with: the perceived depressiveness of PALS settled on NIV (rho = −.180, *p <* .001), IV (rho = −.232, *p <* .001), and PEG (rho = −.184, *p <* .001); perceiving IV (rho = −.189, *p <* .001) and PEG (rho = −.114, *p =* .018) as beneficial measures for PALS; and taking an ideatory decision in favor of PEG (rho = −.101, *p =* .036) and/or IV by neurologists in case of indications in their own cases. In addition, the estimated QoL on PEG (rho = .112, *p =* .025) correlated positively with an attitude toward the termination of an LSM.

*Shared‐decision approach*: A positive attitude toward withdrawing an LSM correlated with a favorable declaration of greater patient autonomy in the shared decision‐making process (rho = .179, *p <* .001).

*Emotional burden*: There was a negative correlation between the perceived emotional distress and the LSM ceasing attitude (rho = −.150, *p =* .002).

*Cross‐national differences*: German neurologists reported a more favorable approach toward halting an LSM as compared to the Polish (CI 95%, .96–1.80, *p <* .001) (“Yes”/“Rather Yes”: 16.1%/57.6% vs. 11.6%/24.6%, respectively). German neurologists with completed PCT (CI 95%, .45–2.18, *p =* .003) and Polish male neurologists (CI 95%, .01–1.13, *p =* .046) were more prone to express a willingness to withdraw LSM.

#### 3.4.2 “Do you think ALS patients ask their physicians to implement an exit strategy because of a low QoL/decreased mood/being a burden to their families/closest ones?”

In 70.5%, 50.1%, and 48.8% of cases, the surveyed neurologists stated a low QoL, a willingness to lessen the burden on the family/closest ones, and decreased mood, respectively, were most likely the primary reasons to wish for hastening the death among PALS.

*Demographic and clinical predictors*: Lack of PCT was strongly associated with declaring a low QoL as the reason for the wish to hasten the death among PALS (OR: 1.82 [1.24–2.68], *p =* .002), whereas higher values of self‐reported religiousness were related to pointing the decreased mood (OR: 1.29 [1.02–1.63], *p =* .033) as the cause.

A link between LSM and ES/shared‐decision approach/emotional burden: The answers were not found related to the views on LSM, shared decision‐making, and emotional burden.

*Cross‐national differences*: German and Polish neurologists presented parallel rates in responses (a *low QoL*: 72.8% vs. 68.4%, respectively; *p =* .292, a *burden on the family/closest ones*: 53.5% vs. 46.8%, respectively; *p =* .150, and *decreased mood*: 50% vs. 47.7%, respectively; *p =* .617). Unlike the Poles, the Germans who quoted *a low QoL* reported more profound emotional distress (*p =* .031), whereas those who cited *a depressed mood* were less likely to contribute to the decision‐making process (*p =* .019).

#### 3.4.3 “Should euthanasia be legalized in your country?”

Of the surveyed neurologists, 63.6% opposed the legalization of euthanasia, with 35.2% answering *No*, 28.4% *Rather no*, 22.6% *Rather yes*, and 13.8% *Yes*.

*Demographic and clinical predictors*: Neurologists of *younger age* (*p =* .004) and those with *lower values of self‐reported religiousness* (*p <* .001) had a more positive approach toward the legalization of euthanasia.

*A link between LSM and ES*: Defining PEG and IV as beneficial measures for PALS correlated inversely with the favorable approach to euthanasia legalization (rho = −.122, *p =* .012, and rho = −.102, *p =* .035, respectively). The same relationship concerned neurologists’ hypothetical decision to opt for PEG or IV in case of medical indication (rho = −.130, *p =* .007, and rho = −.110, *p = .023*, respectively).

*Shared‐decision approach*: A positive correlation was observed between advising on medical matters and a favorable stance toward legalizing euthanasia (rho = .107, *p =* .029).

*Cross‐national differences*: German and Polish neurologists demonstrated similar rates of opposition toward the idea of legalizing euthanasia (64.8% and 62.6%, *p =* .601). However, Polish participants were more likely to express a clear stance on the issue, with 54.2% providing a definite “Yes” or “No” compared to 43.5% of Germans (*p <* .001). Table 6 shows additional differences between the two countries.

**TABLE 6:** Relationship between beliefs about percutaneous endoscopic gastrostomy (PEG), non‐invasive ventilation (NIV) and invasive ventilation (IV) and the attitude toward euthanasia legalization: German and Polish neurologists.

	Positive attitude toward euthanasia legalization	
	Germany	Poland
*“Do you consider PEG a beneficial measure for PALS?”^1^*	−.066	−.145^a^,**
*“Do you consider NIV a beneficial measure for PALS?” ^1^*	−.042	−.089
*“Do you consider IV a beneficial measure for PALS?” ^1^*	−.063	−.175^a^,**
*“Would you decide for PEG yourself in case of indication?” ^1^*	−.148^a^,**	−.087
*“Would you decide for NIV yourself in case of indication?” ^1^*	−.153^a^,*	.003
*“Would you decide for IV yourself in case of indication?” ^1^*	−.042	−.224^a^,**
*“Should physicians advice patients on LSM?” ^1^*	.015	.220^a^,**
*“Would you withdraw the LSM when asked by a patient?” ^1^*	.117	.455^a^,**

Abbreviations: LSM, life‐sustaining measures; PALS, patients with Amyotrophic Lateral Sclerosis.

**^a^ Significant at an alpha‐level of p < .05**

* *p* < .05.

** *p* < .01.

## 4 DISCUSSION

### 4.1 General practice

The survey shows that one in every three neurologist initiates the discussion regarding ESs (euthanasia or PAS) with ALS patients, with similar rates across both surveyed countries. Typically, these conversations begin during the advanced stage of the disease. This observation is noteworthy for several reasons. One is the lack of difference in the ratings between the two countries, despite a considerable dissimilarity in local law concerning PAS. Second, considering that any practices explicitly intended to shorten life are equated to homicide in Poland, the observed approximate ratio of 1:2 of Polish neurologists who trigger the ESs discussions with ALS patients, compared to those who do not bring this topic up, is strikingly high. The results suggest that physicians lean more toward their personal, professional, and culture‐based approaches, rather than strictly adhering to the legal bases, when engaging in discussions with patients. In addition, neurologists, likely to underrate the QoL in patients with ALS (Aho‐Özhan et al., 2017; Barć et al., 2022), may choose to familiarize the patient with all, even hypothetical, procedures of palliative care. This can result in significant variations in the frequency and content of these discussions, even within the same locations. Therefore, the introduction of specific guidelines could help to reduce these inconsistencies. However, these findings should be interpreted carefully. A prior study showed that physicians’ hypothetical statements about end‐of‐life care may not consistently reflect their actual/future practices (Abrahao et al., 2016).

#### 4.1.1 Demographic and clinical determinants

Predictors such as younger age, male gender, and lower values of self‐reported religiousness were related to a higher eagerness to commence the conversation on ESs. In both analyzed countries, neurologists presenting a more decisive approach to the decision‐making process and those with lower values of emotional distress more commonly began the discussion. It shows that a confident attitude of the physician may play an influential role in the patient's decision‐making process as it may facilitate addressing the issues of terminal care. Our preceding paper concerning LSMs reported a similar relationship (Barć et al., 2022).

Previous publications showed that higher professional experience of physicians in ALS was likely to result in a more accurate estimation of the well‐being in ALS patients who used LSMs (Aho‐Özhan et al., 2017; Barć et al., 2022). It was presumably related to a higher awareness of the patient's perspectives on end‐of‐life treatment. In addition, higher perceptions of the well‐being of individuals using LSMs lead to earlier introduction of these measures to patients (Barć et al., 2022). The current study provides additional evidence for the importance of estimates of well‐being in relation to initiating a dialog concerning ES. Specifically, neurologists who perceive that patients have satisfactory well‐being while on LSMs are more likely to take up the conversation about ES. This suggests that these physicians want to increase the patient's awareness of all options for palliative care, including those aimed at prolonging or shortening life. This might be especially true for those designating autonomy as the primary goal of their treatment (Craig & Dzeng, 2018; Kono et al., 2023; Mohammed et al., 2020).

### 4.2 Personal experience

A mere 11% of physicians reported ever receiving requests to discontinue LSMs, and fewer than 10%—to implement an ES. Notably, despite a contrasting difference between the legal position of PAS in Germany and Poland, physicians in both countries reported similar rates of requests to terminate an LSM. It has previously been shown that most patients benefit from LSMs (Barć et al., 2020) and change to positive attitudes toward these measures in early‐to‐midst ages of the disease. Nevertheless, specific subsets of people with ALS alter their preferences over the long term, opting not to utilize these options (Hayashi & Oppenheimer, 2003; Mitsumoto et al., 2005; Ozeki‐Hayashi et al., 2022) in the diseases’ advanced stages, partially due to a general loss of meaning in life (Dreyer et al., 2012).

Contrary to their Polish counterparts, German neurologists more commonly received requests to employ an ES. Given that both countries were comparable in introducing ESs to patients with ALS, this difference may be attributed to other factors, such as advance directives (obligatory to obey by German physicians, while not legally binding in Poland) or variations in the content of PCT in the respective country (Barć et al., 2022; Pawłowski et al., 2019; Wiesing et al., 2010).

### 4.3 A personal approach

The willingness of neurologists to implement patients’ desired end‐of‐life care measures is equally important as the patients’ wishes themselves. In our cohort, German neurologists demonstrated a greater inclination to support the termination of LSMs compared with their Polish counterparts (73.7% vs. 36.2%). This primarily reflects the fundamental differences in national laws concerning PAS. Nonetheless, despite these discrepancies, approximately one third of Polish neurologists expressed readiness to discontinue LSMs in response to a patient's request. This stance highlights the willingness of some physicians to address end‐of‐life issues, even if it entails legal implications, potentially stemming from their respect for patient autonomy as a guiding principle (Craig & Dzeng, 2018; Kono et al., 2023). Our questionnaire did not concurrently explore the neurologists' hypothetical decisions regarding euthanasia or PAS implementation. In a 2015 report, the authors surveyed 192 German physicians and discovered that a mere 5.3% of respondents declared a willingness to perform euthanasia, whereas 79.6% rejected the notion (Zenz et al., 2015). The relatively low approval rate among this group indicates that personal beliefs pertaining to the withdrawal of LSMs and the implementation of ESs are influenced by diverse backgrounds.

According to the survey results, two thirds of neurologists believed that a low QoL, and for approximately 50%, a decreased mood could be the hypothetical reasons for ALS patients to request an ES. This view contrasts sharply with the patients’ perspectives on this issue, where neither the QoL nor depressed mood affects requesting or not an ES (Lulé et al., 2014; Maessen et al., 2014). This disagreement between the views of patients and physicians may raise serious concern in the decision‐making process, as the opinions of neurologists are likely to contribute to the preliminary decision made by patients (Barć et al., 2022).

Euthanasia has been a contentious subject of debate worldwide, with personal characteristics such as male gender, younger age, higher social class, higher education, and lower values of self‐reported religiousness leading to greater approval of the legalization of euthanasia in the general population (Brinkman‐Stoppelenburg et al., 2020; Cohen et al., 2006; Inglehart et al., 2021). In Europe, attitudes toward euthanasia acceptance vary widely between nations, with the Netherlands and Switzerland showing a more favorable reception than traditionally Catholic countries like Poland (Cohen et al., 2006; Inglehart et al., 2021). Moreover, although there has been an increase in euthanasia acceptance in recent years in most countries (such as Norway, Germany, and Poland), there are still some countries that have experienced a decline in acceptance (such as Greece) (Cohen et al., 2006; Inglehart et al., 2021). Approval rates for euthanasia endorsement are generally lower among physicians than in the general population. This is especially true for female individuals and those with religious beliefs (Brinkman‐Stoppelenburg et al., 2020; Evenblij et al., 2019; Zenz et al., 2015). Furthermore, the acceptability of PAS depends on the underlying condition that is the cause for the demand, with physical disorders being the most commonly accepted (Bolt et al., 2015). In our cohort, the majority of German and Polish neurologists opposed the legalization of euthanasia (64.8% and 62.6%, respectively). In both countries, higher levels of religiosity were associated with lower acceptance of euthanasia, confirming a previous report on physicians’ perspectives from European ALS centers (Thurn et al., 2019). In addition to religiousness, younger age and lower emotional burden were found to be positively correlated with euthanasia acceptance. This finding is consistent with other studies (Ashraf et al., 2022) but reveals this interplay for the first time in the context of ALS.

Previous studies have shown a negative relationship between completed training in palliative care and a positive approach to executing an ES (Marini et al., 2006; Zenz et al., 2014). However, a recent report analyzing physicians’ practices in caring for patients with ALS showed no influence of training in palliative care on PAS but noted a more liberal attitude toward continuous sedation until death and withdrawal of IV (Thurn et al., 2019). In our cohort, neurologists who received training in palliative care reported more frequent requests to terminate LSMs and to implement ESs for ALS patients. This suggests that physicians with a deep understanding of end‐of‐life care may have greater self‐confidence and are more likely to address challenging topics of terminal care with patients in advanced disease stages. Including palliative care specialists appears to be essential to improve the quality of terminal care for patients with ALS (Pelayo et al., 2011).

## 5 LIMITATIONS

The major limitation of the current study is the low response rate that may have affected the study outcomes. Neurologists lacking or without experience in ALS were most likely to decline participation in the study, suggesting that our results may be more applicable to those who routinely manage ALS patients, rather than the general neurologist population. Due to the anonymous nature of the survey, sending a reminder was impossible. Yet of note, based on the given replies, physicians from all regions in Germany and Poland partook in the survey. Furthermore, similar response rates have been reported for other surveys following similar procedures.

## 6 CONCLUSION

Although the majority of neurologists do not endorse euthanasia legalization, approximately one third engage in ES discussions with ALS patients, and an additional 50% are amenable to addressing such requests. Factors influencing participation in these dialogs include demographic characteristics (male gender, younger age), personal values (religious beliefs), professional experience (PCT training and involvement in decision‐making processes), as well as patients’ emotional burden, and ideatory QoL related to PEG, NIV, and IV use. Despite this, a mere 11% of surveyed physicians reported having received requests to withdraw LSMs and only 10% for implementing life‐ending strategies, corroborating prior research findings that ALS patients tend to prioritize living over hastening death (Kuzma‐Kozakiewicz et al., 2019; Lulé et al., 2014; Lulé et al., 2009).

## AUTHOR CONTRIBUTIONS

**Krzysztof Barć**: conceptualisation (equal); data curation (lead); formal analysis (lead); investigation (equal); methodology (equal); writing—original draft (lead); writing—review and editing (lead). **Dorothée Lulé**: conceptualisation (equal); data curation (equal); funding acquisition (lead); methodology (equal); project administration (equal); supervision (lead); writing—review and editing (equal). **Julia Finsel**: investigation (supporting); resources (supporting); writing—review and editing (supporting). **Olga Helczyk**: investigation (supporting); resources (supporting); writing—review and editing (equal). **Susanne Baader**: investigation (supporting); resources (supporting); writing—review and editing (equal). **Helena Aho‐Oezhan**: investigation (equal); resources (equal); writing—review and editing (equal). **Albert C Ludolph**: project administration (equal); supervision (equal); validation (equal); writing—review and editing (equal). **Magdalena Kuźma‐Kozakiewicz**: conceptualisation (equal); funding acquisition (lead); investigation (equal); methodology (equal); project administration (lead); resources (lead); supervision (lead); validation (equal); writing—original draft (equal); writing—review and editing (equal).

## INFORMED CONSENT

Each participant was informed of the purpose of the study in the introduction letter provided with the questionnaire. Filing the anonymous questionnaire form was considered informed consent of participation.

## CONFLICT OF INTEREST STATEMENT

The authors declare that there are no conflicts of interest that could be perceived as prejudicing the impartiality of the research reported.

### PEER REVIEW

The peer review history for this article is available at https://publons.com/publon/10.1002/brb3.3243.

## Supporting information

Supplementary material A: Components Addressed in the Questionnaire

Supporting Information

## Data Availability

The data that support the findings of this study are available from the corresponding author upon reasonable request.
