# Supplementary material for: One third of physicians discuss exit strategies with patients with amyotrophic lateral sclerosis: Results from nationwide surveys among German and Polish neurologists
Source: Brain Behav. 2024 Jan 6;14(2):e3243. doi: 10.1002/brb3.3243 (PMC10897500; doi:10.1002/brb3.3243)
Supplement: Supplementary file 1 — Supplementary material A: Components Addressed in the Questionnaire [file BRB3-14-e3243-s001.docx]

**Supplementary material A: Components Addressed in the Questionnaire**

*General practice.* We asked physicians about their approach to the discussion of ES with PALS. The choices were the following: (i) “I initiate such discussion”: (a) at the disease diagnosis, (b) at an advanced stage of the disease), (ii) “I discuss it only when asked by a patient”, (iii) “I have never attempted such conversation” (there was no occasion to do so *or* refusal of the discussion despite the patient’s request).

*Personal experience.* The respondents were questioned to indicate whether they had ever been asked to (i) terminate an LSM, and to (ii) implement an ES, with binary options of “Yes” and “No”.

*Attitudes towards ES.* Neurologists were also asked to quote the principal causes of a wish for hastened death among PALS and to declare their personal opinion on euthanasia legalization (“Yes”, “Rather yes”, “Rather no”, “No”).

*Demographic and clinical predictors.* We investigated a potential relationship between demographic and work experience variables and all the answers.

*A link between LSM and ES.* To study the possible interplay between the thoughts on LSM and ES, physicians were asked to (i) estimate the QoL of PALS using (1a) PEG, (1b) NIV, and (1c) IV with an adapted version of the anamnestic comparative self-assessment (ACSA), on a Likert scale ranging from -5 (as bad as possible) to 5 (as good as possible), (ii) estimate the depressiveness on each of these measures on a Likert scale ranging from 0 (no symptoms of depression) to 10 (severe symptoms of depression), (iii) define each of these LSM as beneficial to PALS (“Yes”, “Rather yes”, “Rather no”, “No”), and (iv) indicate the hypothetical decision to use a certain LSM by themselves in case of medical indications (“Yes”, “Rather yes”, “Rather no”, “No”).

*Shared decision approach.* To determine the physicians’ contribution to the decision-making process, all respondents were asked to (i) choose one of five attitudes on the adapted version of the “Decision Shared Making Questionnaire”, ranging from the decision solely made by the physician (1), to mutual contribution of both sides (3), and the decision being left to the patent alone (5). For details, see **Table I,** **Supplementary materials B**. They were also requested to (ii) decide whether the physician should advise on therapeutic choices to a patient (“Yes”, “Rather yes”, “Rather no”, “No”).

*Emotional burden.* We asked respondents (i) whether they agreed that caring for PALS was emotionally distressing for them, based on the following options: “Yes”, “Rather yes”, “Rather no”, “No”.

*Cross-national differences.* All of these aspects were additionally analyzed separately for each country.
