# Supplementary material for: One third of physicians discuss exit strategies with patients with amyotrophic lateral sclerosis: Results from nationwide surveys among German and Polish neurologists
Source: Brain Behav. 2024 Jan 6;14(2):e3243. doi: 10.1002/brb3.3243 (PMC10897500; doi:10.1002/brb3.3243)
Supplement: Supplementary file 2 — Supporting Information [file BRB3-14-e3243-s002.docx]

**Supplementary materials B, Table I.** Shared Decision Questionnaire used to evaluate the physician’s role in the decision-making process

| “Decision shared questionnaire” | |
| --- | --- |
| 1. | *Decisions should be made solely by the physician* |
| 2. | *Decisions should be made by the physician, but with consideration of the patient's opinion* |
| 3 | *Decisions should be made mutually - by the physician and the patient* |
| 4. | *Decisions should be made by the patient, but with consideration of the physician's opinion* |
| 5. | *Decisions should be made solely by the patient* |

**Supplementary materials B, Table II.** Neurologists’ opinions about exit strategies in patients with amyotrophic lateral sclerosis

|  | **Entire population** | **German population** | **Polish**  **population** | *p-value* |
| --- | --- | --- | --- | --- |
| “Do you discuss exit strategies (*euthanasia* or *physician-assisted suicide*) with ALS patients?” (%) | | | | |
| (i) initiate  a) at the diagnosis  b) at later stages  (ii) only on a request  (iii) never | 30.2  5.3  24.9  47.0  22.8 | 32.9  8.2  24.7  56.6  10.5 | 27.5  2.4  25.1  37.0  35.5 | *<0.001******** |
| “Have you ever been asked to terminate a life-sustaining measure?” (%) | | | | |
| Yes  No | 11.0  89.0 | 13.4  86.6 | 8.8  91.2 | *0.131* |
| “Have you ever been asked to implement an exit strategy?” (%) | | | | |
| Yes  No | 9.9  90.1 | 17.0  83.0 | 3.1  96.9 | *<0.001******** |
| “Would you terminate a life-sustaining measure if asked by a patient?” (%) | | | | |
| No  Rather no  Rather yes  Yes | 21.1  24.3  40.8  13.8 | 4.1  22.1  57.6  16.1 | 37.5  26.3  24.6  11.6 | *<0.001******** |
| “Do you think patients with ALS ask their physicians to implement an exit strategy because of a low QoL?” (%) | | | | |
| Yes  No | 70.5  29.5 | 72.8  27.2 | 68.4  31.6 | *0.292* |
| “Do you think patients with ALS ask their physicians to implement an exit strategy because of a depressed mood?” (%) | | | | |
| Yes  No | 48.8  51.2 | 50.0  50.0 | 47.7  52.3 | *0.617* |
| “Do you think patients with ALS ask their physicians to implement an exit strategy because of being a burden to their families/closest ones?” (%) | | | | |
| Yes  No | 50.1  49.9 | 53.5  46.5 | 46.8  53.2 | *0.150* |
| “Should euthanasia be legalised in your country?” (%) | | | | |
| No  Rather no  Rather yes  Yes | 35.2  28.4  22.6  13.8 | 32.4  32.4  24.1  11.1 | 37.9  24.7  21.1  16.3 | *0.119* |

**ALS** = amyotrophic lateral sclerosis; **ES** = exit strategy (*euthanasia* or *physician-assisted suicide*); **QoL** = quality of life. *****Statistically significant difference between the two countries.

**Supplementary materials B, Table III.** Demographic and clinical predictors of neurologists’ perspective on exit strategies in patients with amyotrophic lateral sclerosis: multiple regression approaches

|  | “Do you discuss exit strategies (*euthanasia* or *physician-assisted suicide*) with ALS patients?” | “Have you ever been asked to terminate a life-sustaining measure?” | “Have you ever been asked to implement an exit strategy?” | “Would you terminate a life-sustaining measure if asked by a patient?” | “Do you think PALS ask their physicians to implement an ES because of a low QoL?” | “Do you think PALS ask their physicians to implement an ES because of depressed mood?” | “Do you think PALS ask their physicians to implement an ES because of being a burden to their families/closest ones? | “Should euthanasia be legalised in your country?” |
| --- | --- | --- | --- | --- | --- | --- | --- | --- |
| *demographic* |  |  |  |  |  |  |  |  |
| **age** | ✔ |  |  | ✔ |  |  |  |  |
| **gender** | ✔ |  |  |  |  |  |  |  |
| **religiousness** | ✔ |  |  | ✔ |  | ✔ |  | ✔ |
| **children** |  |  |  |  |  |  |  |  |
| **relationship** |  |  |  |  |  |  |  |  |
| **nationality** | ✔ | ✔ | ✔ | ✔ |  |  |  | ✔ |
| *clinical* |  |  |  |  |  |  |  |  |
| **ExR** |  |  |  |  |  |  |  |  |
| **PCT** |  | ✔ | ✔ |  |  |  |  |  |
| **ALS research** |  | ✔ |  |  | ✔ |  |  |  |

**ALS** = amyotrophic lateral sclerosis. **ExR** = Experience Rate (calculated as: a *number* of patients with amyotrophic lateral sclerosis seen per month on average * a *number* of years in experience in ALS). **PCT** = Palliative care training. ✔ - variable is statistically significant.
